# 2522. Antisense Antibiotics are Effective Against *Pseudomonas aeruginosa* Biofilm

**DOI:** 10.1093/ofid/ofad500.2140

**Published:** 2023-11-27

**Authors:** Paul J Merville, Garrett Ray, David E Greenberg, Christine A Pybus, Ibrahim Helaibi

**Affiliations:** University of Texas Southwestern Medical Center, Dallas, Texas; University of Texas Southwestern Medical Center, Dallas, Texas; UT Southwestern, Dallas, Texas; UT SOUTHWESTERN MEDICAL CENTER, DALLAS, TX; Alabama State University, Montgomery, Alabama

## Abstract

**Background:**

*P. aeruginosa* (*PA*) causes severe infections in immunocompromised patients and is increasingly antibiotic resistant. In addition, the formation of biofilm by *PA* makes effective treatment difficult. Peptide-conjugated phosphorodiamidate morpholino oligomers (PPMOs) are novel antisense antimicrobials that are designed to target pathogens in a gene-specific way and prevent translation of protein. We have demonstrated *in vitro* and *in vivo* activity of lead PPMOs in *PA*; however, it remains unclear whether PPMOs remain active in the biofilm setting over time and if synergy can be achieved with traditional antibiotics.

**Methods:**

*P. aeruginosa* (GFP-PAO1) was dosed with a rhodamine-labelled PPMO targeting *acpP*. Samples were fixed at different time intervals and imaged by fluorescent microscopy. A Pearson’s coefficient was recorded for each dose and time condition. Minimum biofilm eradication concentration plates were used to treat *PA* biofilm with *acpP* or *rpsJ* targeted PPMOs in daily dosing experiments. Fixed synergy assays were performed on mature biofilm via 24-hour Q8 dosing with PPMO, antibiotics or control. Biofilm burden was quantified by CFU enumeration.

**Results:**

PPMOs co-localized with *PA* in a dose- and time-dependent fashion. Pearson coefficients increased from 0.0583 at the 30-minutes to 0.4118 and 0.4976 at the 3- and 5-hour time points respectively. In addition, biofilm reduction was dose-dependent. In daily dosing experiments, the 3’RXR4-AcpP PPMO resulted in a 5-log reduction in biofilm burden compared to control on day 7 [*p< 0.0001*]; the 5’RXR4-RpsJ PPMO resulted in a 5.5-log reduction in biofilm burden compared to no treatment control [*p< 0.0001*]. PPMOs incubated with tobramycin or aztreonam were found to have synergistic (FIC< 0.5) or antagonistic effects (FIC > 1) in biofilm and planktonic killing depending on the combination tested.

**Conclusion:**

Lead *PA* PPMOs demonstrate dose and time-dependent biofilm eradication over multiple days of therapy. In addition, some PPMOs demonstrate synergy with other small molecule antibiotics that are used to treat certain patients with *PA* infections. *PA* PPMOs could be an innovative treatment modality for infection due to *Pseudomonas*.

**Disclosures:**

**David E. Greenberg, MD**, University of Texas Southwestern Medical Center: Dr. Greenberg has numerous patents on PPMOs

